# The Impact of Internet and Mobile Phone Usage and Unemployment on Adult Obesity: Empirical Evidence from the BRICS States

**DOI:** 10.3390/healthcare13212765

**Published:** 2025-10-30

**Authors:** Gamze Sart, Yilmaz Bayar, Marina Danilina, Marius Dan Gavriletea

**Affiliations:** 1 Department of Educational Sciences, Hasan Ali Yucel Faculty of Education, Istanbul University-Cerrahpaşa, 34500 İstanbul, Turkey; gamze.sart@iuc.edu.tr; 2 Department of Public Finance, Faculty of Economics and Administrative Sciences, Bandirma Onyedi Eylul University, 10200 Bandirma-Balikesir, Turkey; 3 Department of Economics, Plekhanov Russian University of Economics (PRUE), 117997 Moscow, Russia; marinadanilina@yandex.ru; 4 Department of Economics, Financial University under the Government of the Russian Federation, 125167 Moscow, Russia; 5 Department of Business, Business Faculty, Babeş-Bolyai University, 400174 Cluj-Napoca, Romania; marius.gavriletea@ubbcluj.ro

**Keywords:** adult obesity, Internet usage, mobile phones, unemployment, panel data analysis

## Abstract

**Background/Objectives**: The number of overweight and obese people has significantly increased in the world, and this phenomenon is referred to as globesity. Globally increasing obesity has become one of the major problems to be dealt with for countries, given obesity-related health problems, including nutrition-related noncommunicable diseases and some types of cancer, and the economic and social costs of obesity. Therefore, countries try to combat obesity through diverse strategies related to nutrition, physical activity, and education. In this regard, identifying the factors behind obesity is critical to making progress in the fight against obesity. **Methods**: This study explores the interplay amongst ICT (information and communication technologies) indicators, including Internet and mobile phone usage, unemployment, and adult obesity in the BRICS states from 1995 to 2022, using recently developed cointegration techniques and causality tests. **Results**: The outcomes of causality tests uncover an interaction between Internet and mobile phone usage, unemployment, and adult obesity. In addition, the cointegration coefficients reveal that Internet and mobile phone usage positively impact adult obesity, while unemployment has a negative effect on adult obesity. **Conclusions**: Our outcomes uncover that improper use of the Internet and mobile phones foster adult obesity, but proper utilization of the Internet and mobile phones can be effective instruments in combatting adult obesity through increasing the awareness of healthy lifestyles and online weight loss programs.

## 1. Introduction and Background

Obesity and overweight have been accepted as one of the most important public health problems for all age groups, and have been amongst the global priorities to be addressed. Hence, the WHO (World Health Organization) states that obesity among adults has more than doubled globally as of 1990, and obesity among adolescents has quadrupled during the same period [1]. The World Obesity Federation also says that there have been nearly 3 billion overweight and obese people in the world [2]. Furthermore, NCD Risk Factor Collaboration estimates that nearly 880 million adults and 159 million children and adolescents aged 5–19 years are living with obesity [3].

Obesity has many health- and economic-related implications for society. In this respect, firstly, obesity may cause noncommunicable diseases such as cancers, diabetes, cardiovascular diseases, chronic respiratory diseases, digestive disorders, and neurological disorders [4], and nearly 1.6 million people die annually, owing to the non-communicable diseases caused by obesity and overweight [5]. Secondly, obesity also has economic costs related to reduced or lost productivity and human capital [6]. Thirdly, considerable public and private funds are allocated to the treatment of obesity-related illnesses. Consequently, the global economic cost of obesity and overweight is projected to reach US $4.32 trillion per year by 2035 if effective preventive and remedial measures are not implemented [7].

Obesity is a global public health issue, and its worldwide prevalence continues to rise. It primarily results from an imbalance between dietary intake and physical activity [1]. However, factors such as insufficient income and education, which limit access to healthy food, opportunities for physical activity, and health services, can also contribute to obesity through pathways such as undernutrition and inadequate healthcare [8]. Despite this, Islam et al. [9] found that the prevalence of obesity was relatively higher among individuals with higher income. Similarly, Ameye and Swinnen [10] reported that obesity was typically more prevalent among rich individuals in low-income countries, and among poorer individuals in high-income countries. Therefore, further empirical research is required to uncover the underlying causes of obesity in countries with varying social and economic characteristics. Identifying these factors would also aid policymakers in designing and implementing effective preventive measures to manage and reduce obesity.

In the empirical literature about the determinants of obesity, Islam et al. [9] suggested a significant relationship between overweight/obesity and economic status, as a result of meta-regression on 73 studies. On the other hand, Ameye and Swinnen [10] found a non-linear relationship between income and obesity in a global context. Daran et al. [11] revealed a positive connection between socio-economic status and obesity in low-income sub-Saharan African countries, but a mixed relationship between the two variables in lower-middle-income countries. Raftopoulou and Gil Trasfi [12] uncovered the presence of income-related inequalities in obesity in Spain. Last, the extensive literature review of Jalilzadeh and Goharinezhad [13] indicated that income was a significant determinant of obesity.

The research of Jalilzadeh and Goharinezhad [13] and Lee et al. [14] suggested that the demographic factors such as age, gender, race, and ethnicity were significant factors underlying obesity. On the other hand, Żukiewicz-Sobczak et al. [8] specified less education as a determinant of obesity amongst poor people, and Raftopoulou and Gil Trasfi [12] also suggested that equal access to higher education would make a contribution toward reducing the income-related inequality in obesity. Additionally, Żukiewicz-Sobczak et al. [8] and the research of Jalilzadeh and Goharinezhad [13] indicated that unemployment was a significant factor of obesity. Lee et al. [14] and Żukiewicz-Sobczak et al. [8] also emphasized that nutrition, low physical activity, and the use of alcohol and cigarettes are significant drivers of obesity. Last, Jalilzadeh and Goharinezhad [13] and Sart et al. [15] identified technological progress and globalization as being the factors underlying obesity.

This study explores the impact of Internet, mobile phone usage, and unemployment on adult obesity in the BRICS states, given the presence of few studies on the drivers of obesity. On the one hand, Internet and mobile phone usage enables people to access information about health, healthy nutrition [16] and diet, physical activity, and fitness [17,18]. Furthermore, people can access information anytime and anywhere, and Internet-based programs also provide anonymity to the individuals avoiding face-to-face treatment [19]. On the other hand, the usage of the Internet and mobile cellular telephones can positively impact obesity through changing a person’s diet in an unhealthy way, less physical activity [20], low sleep quality, and worsened mental health [21]. In conclusion, the net impact of Internet and mobile phone usage on obesity can differ based on how individuals utilize the Internet and mobile phones.

In similar vein, the related empirical studies about the relationship between Internet usage and obesity have found different results. Beleigoli et al. [22] and Aghasi et al. [23] revealed a positive relationship between Internet usage and the odds of being obese as a conclusion of their literature review. However, Liu et al. [18] and Souza et al. [24] suggested that Internet usage improved healthy nutrition and physical activity. Shi et al. [25] also revealed a significant effect of Internet-based programs on weight loss as a result of the literature review.

Furthermore, unemployment can positively impact obesity through preventing people from accessing healthy foods, instruments of physical activity, and healthcare services by way of income channel. On the other hand, psychological problems resulting from unemployment can also affect obesity through an unhealthy lifestyle, alcohol usage, smoking, low sleep quality, and irregular nutrition [26,27]. Furthermore, obesity can reduce the employment possibility of unemployed people through low productivity and sick leave, resulting from health problems or social discrimination [28,29].

The number of obese people (percentage of adults aged 18 and older with a BMI (body mass index) of 30 kg per square meter or higher) has remarkably increased in all BRICS (Brazil, Russian Federation, India, China, and South Africa) countries between 1995 and 2022. The increases in the obesity rate in India, China, Brazil, South Africa, and the Russian Federation were 507.353%, 383.093%, 173.484%, 58.164%, and 39.942%, respectively [30]. On the other hand, these countries have experienced abnormal increases in both Internet and mobile phone usage during the same period. In this context, the increases in Internet usage were 1,527,172.727% (China), 213,258.779% (India), 76,566.667% (Brazil), 60,981.081% (Russian Federation), and 11,052.142% (South Africa), while the increase in mobile cellular telephone subscriptions per 100 people were 284,781.514% (Russian Federation), 1,014,085.482% (India), 41,817.574% (China), 13,662.783% (South Africa), and 12,478.594% (Brazil) [31,32]. Therefore, this study investigates the role of significant increases in ICT indicators, along with unemployment, on adult obesity, considering the above-mentioned theoretical views and empirical results.

In light of the above-mentioned theoretical and empirical considerations, this study delves into the relationship between Internet and mobile phone usage, unemployment, and obesity in the BRICS states from a macro perspective, which is different from previous studies investigating the relationship between ICT indicators and obesity from the micro perspective. Furthermore, the researchers have concentrated on the impact of ICT indicators and unemployment on obesity in the associated empirical literature, but the impact of obesity on ICT usage has been ignored to a great extent. Therefore, utilization of the causality test in this article also allows us to perform a two-way analysis between Internet and mobile phone usage, unemployment, and obesity. The causality test makes it possible to examine the effect of obesity on Internet and mobile phone usage and unemployment.

The structure of this article is organized as follows. The subsequent section provides a review of the limited empirical literature on the impact of Internet and mobile phone usage on obesity and overweight. This is followed by a brief introduction to the dataset and methodological approach. Section 4 presents the econometric analyses and discusses the empirical results. Finally, the article concludes with a summary of key findings and policy implications.

## 2. Literature Overview

Obesity has become a crucial problem for both developed and developing countries, and remedial and preventive measures should be implemented to address its negative health, social, and economic implications. Despite the growing body of literature on the determinants of obesity and overweight, empirical investigations into the multifaceted relationship between Internet and mobile phone usage, unemployment, and obesity remain relatively scarce. Within this limited literature, the nexus between Internet usage and obesity has been examined by a small number of scholars, who have predominantly identified a positive association for different countries. In this regard, Eliacik et al. [33], Yıldız et al. [34], Ma and Sheng [35], Deng et al. [36], Guo et al. [21], Liu et al. [37], and Azizi et al. [38] discovered a positive connection between diverse indicators of Internet usage and obesity for the developing countries of Turkey, China, and Iran, while Lin et al. [39] found a positive relationship between high-speed Internet access and obesity in Australia. On the other hand, Liu et al. [18] and Belojevic et al. [40] have identified a negative association between Internet usage and obesity in more highly educated Chinese males in urban regions and Sombor, a small district of Serbia, respectively.

Eliacik et al. [33] investigated the relationship between Internet addiction and obesity among 71 adolescents diagnosed with obesity at Tepecik Teaching Hospital and Katip Çelebi University Hospital in İzmir, Turkey. Their findings indicated that Internet addiction and reduced physical activity were associated with an increased likelihood of obesity. Similarly, Yıldız et al. [34] examined the relationship between Internet addiction and obesity among first-year university students at Dokuz Eylül University in Turkey and reported a positive association between the two variables.

Ma and Sheng [35] examined the impact of Internet usage on BMI among Chinese adolescents over the period 2004–2015 using regression analysis, and reported a positive impact of Internet usage on BMI. Similarly, Deng et al. [36] investigated the effect of Internet usage on nutritional intake and health outcomes, using data from rural China. Their findings indicate that while Internet use enhanced dietary knowledge, it also increased the risk of obesity among rural populations. Guo et al. [21] explored the relationship between Internet usage and BMI among the elderly population in China during 2011–2015 and found a positive association between Internet use and BMI. Liu et al. [37] also assessed the impact of Internet use on BMI in a sample of Chinese adolescents, reporting that Internet usage led to an increase in BMI in this group.

Azizi et al. [38] explored the interplay among BMI, Internet addiction, and emotional dysregulation in a sample of adolescents from Tekab, Iran, and revealed a positive impact of Internet addiction on BMI. In a different context, Lin et al. [39] analyzed the relationship between high-speed Internet access and obesity in Australia, uncovering a positive effect of high-speed Internet access on obesity.

However, Liu et al. [18] examined the impact of Internet use on BMI among Chinese males and found a negative impact of Internet usage on BMI, which was particularly pronounced among urban and more highly educated men. Last, Belojevic et al. [40] analyzed the impact of Internet, television and radio usage on BMI among adults in Sombor, Serbia and disclosed a negative impact of Internet on obesity.

The first hypothesis of this study is identified as the following, based on the related theoretical background and the results of the empirical studies for the countries with similar socio-economic development levels:

**H1**: *There is a positive relationship between Internet usage and adult obesity.*

Several studies have investigated the link between mobile phone use and obesity, often identifying a positive interaction across various populations worldwide. In this regard, Akıncı et al. [20], Ma et al. [41], Brodersen et al. [42], and Andersen et al. [43] discovered a positive connection between diverse indicators of mobile phone usage and obesity in different samples from developing and developed countries such as Canada, Denmark, China, and Turkey.

For instance, Ma et al. [41] explored this connection among school-aged children and adolescents in Shanghai, revealing a significant relationship between problematic smartphone use and obesity within this group. Akıncı et al. [20] analyzed the effects of smartphone addiction on obesity and sleep quality in obese men at Haydarpaşa Numune Training and Research Hospital in Turkey, finding a positive association between addiction and obesity. Meanwhile, Brodersen et al. [42] investigated the nexus between smartphone usage and obesity in a sample of 436 young citizen scientists aged 13–21 in Canada and reported that those who spent over 2 h per day on smartphones were three times higher in weight compared to their peers.

Andersen et al. [43] also explored the relationship between nighttime smartphone usage, sleep problems, and obesity and overweight in a Danish adult sample, with results indicating a significant relationship between nighttime smartphone usage and overweight or obesity. Bagheri et al. [44] studied the relationship between smartphone usage and obesity in a sample from Tehran, Iran, revealing a positive correlation between the two.

However, Khokhar et al. [45] conducted a comprehensive literature review, examining the effects of mobile electronic devices, including mobile phones, on weight loss among overweight and obese individuals, concluding that mobile phone interventions significantly facilitate weight reduction. Similarly, Liu et al. [46], Kim and Seo [47], and Pujia et al. [48], through meta-analytical approaches, demonstrated that smartphone-based health programs exert a substantial impact on weight loss outcomes.

Based on the relevant theoretical framework and the empirical evidence, the second hypothesis of this study is formulated as follows:

**H2**: *There is a positive relationship between mobile phone usage and obesity.*

Unemployment can positively impact obesity through diverse channels related to income, psychology, and lifestyle. However, a well-designed social security system can alleviate the negative effects of unemployment on obesity. Several scholars have concentrated on the nexus between unemployment and obesity across various national contexts and have usually found a positive effect of unemployment on obesity. In this regard, Dietrich et al. [29], Aydin and Aydin [27], Latif et al. [49], Hughes and Kumari [50], and Tobing [51] revealed a positive impact of unemployment on obesity in Germany, China, Canada, the UK, the United States of America, respectively.

For example, Dietrich et al. [29] investigated the interaction between obesity and unemployment in Germany by means of logistic regression, and their outcomes revealed that unemployed respondents had a higher probability of being obese. While obesity did not emerge as a determinant of unemployment, obese individuals who were unemployed exhibited a lower probability of re-entering the labor market. Aydin and Aydin [27] also examined the nexus between unemployment and obesity in BRICS countries over the period 1991–2016, using Fourier causality and cointegration tests, and results indicated a significant causality from obesity to unemployment in China.

Latif et al. [49] explored the relationship between macroeconomic indicators, obesity, and BMI in Canada, using regression analysis. Their findings indicated a positive impact of unemployment on the probability of severe obesity and elevated BMI. On the other hand, Hughes and Kumari [50] analyzed the nexus between unemployment and BMI, using data from 10,737 working-age adults in the UK during the 2010–2012 period, and revealed a significant relationship between unemployment and underweight status, as well as a significant relationship between unemployment and obesity in nonsmokers. Lastly, Tobing [51] analyzed the impact of unemployment on obesity in the United States during the Great Recession through regression analysis and indicated a positive effect of unemployment on obesity.

Some researchers have focused on the effect of obesity on unemployment. In this context, Härkönen er al. [52] investigated the impact of obesity on earnings and unemployment in Finland. Their findings demonstrated that obese women faced a higher risk of unemployment compared to their non-obese counterparts, but this relationship was not observed among Finnish men. Similarly, Some et al. [53] analyzed the impact of obesity on unemployment in South Africa, reporting a negative effect of obesity on the likelihood of employment. In a related study, Groves and Wilcox [54] explored the effect of obesity and overweight on the duration of unemployment amongst young American workers, concluding that job-seeking periods were significantly longer for obese and overweight individuals.

Drawing upon the existing theoretical framework and supporting empirical findings, the third hypothesis is proposed as follows:

**H3**: *There is a positive relationship between unemployment and obesity.*

## 3. Materials and Methods

Despite the expanding international evidence, few studies have jointly examined the roles of Internet and mobile phone usage and unemployment in shaping adult obesity within the BRICS economies. Given their distinct combination of economic growth, digital expansion, and social inequality, BRICS countries offer a unique context in which to investigate these relationships. This study contributes to filling this empirical gap by providing a comprehensive macro-level analysis across five major emerging economies.

This study investigates the effects of Internet usage, mobile phone use, and unemployment on adult obesity in the BRICS countries over the period 1990–2022, using cointegration and causality analyses. The variables utilized in the econometric analyses, along with their sources, are reported in Table 1. BMI (body mass index) indicates adult obesity, while INTUSE, MOBUSE, and UNEMP show the Internet and mobile phone usage and unemployment, respectively. In this regard, obesity is proxied by the percentage of adults aged 18 and older with a BMI of 30 kg per square meter or higher, in accordance with the WHO’s definition [1,30]. However, BMI does not sufficiently indicate body fat distribution or distinguish between body fat and lean mass, especially amongst individuals with a BMI < 30 [55]. Internet usage is proxied by the percentage of individuals using the Internet, while mobile phone use is represented by mobile cellular telephone subscriptions per 100 people. Lastly, unemployment is represented by unemployed individuals as a percentage of the total labor force. The ICT indicators of Internet usage and mobile phone use and unemployment are obtained from the World Bank [30,32,56].

The impact of Internet and mobile phone usage and unemployment on adult obesity is investigated for the BRICS countries, and the study period is restricted to within the 1995–2022 period because mobile cellular telephone subscriptions are present for the whole country as of 1995, and all series were available until 2022. Cointegration is performed through Gauss 12.0, cross-sectional dependence (CSD), heterogeneity, unit root, and the causality test, together with the AMG (augmented mean group), which are carried out by means of Stata 17.0.

The descriptive statistics of the study’s variables are reported in Table 2. In this context, the mean values of BMI, INTUSE, MOBUSE, and UNEMP in BRICS countries between 1995 and 2022 are 14.592%, 29.199%, 70.741, and 10.734% per 100 people, respectively. However, the values of the standard deviation indicate that INTUSE and MOBUSE exhibit a noteworthy variation across BRICS nations, while BMI and UNEMP show more moderate differences.

In the context of the methodological approach, CSD and heterogeneity tests are performed through LM (lagrange multiplier) and delta tests, respectively. Then, unit root analysis is carried out by the CIPS unit root test, given the presence of CSD. In addition, the cointegration interplay amongst BMI, INTUSE, MOBUSE, and UNEMP are tested by means of Westerlund and Edgerton’s [57] LM cointegration test, because this test gives reliable outcomes for small datasets and also takes notice of CSD and heterogeneity. Cointegration coefficients of panels and each BRICS state are forecast via the AMG (augmented mean group) estimator suggested by Eberhardt and Bond [58], given the presence of CSD and heterogeneity. Last, the causal relationship between BMI, INTUSE, MOBUSE, and UNEMP is tested by means of the causality test proposed by JKS (Juodis–Karavias– Sarafidis) [59]. The JKS test considers heterogeneity and produces reliable results under the entity of CSD [59]. Additionally, this causality test benefits from the Half Panel Jackknife method by Dhaene and Jochmans [60] to decrease Nickell bias, and thus increases the reliability of the test results. Last, the test provides more robust outcomes for omitted variables than the other causality tests [61].

## 4. Results

In the results and discussion section, the availability of CSD and heterogeneity at the panel dataset are first explored by way of the LM (Lagrange multiplier) and delta tilde tests. Thus, the LMadj., LM CD, and LM tests are carried out, and the H0 hypothesis of CSD independence is rejected because the probability values of these tests, as reported in Table 3, are lower than 1%. Then, delta tilde tests are performed, and the H0 hypothesis of homogeneity is rejected because the probability values of the two delta tilde tests are lower than 5%. The outcomes of the CSD and heterogeneity tests testify the entity of CSD and heterogeneity amongst BMI, INTUSE, MOBUSE, and UNEMP.

The stationarity analysis of the series is required for the selection of the cointegration and causality tests. For this reason, the CIPS unit root test is carried out to examine whether BMI, INTUSE, MOBUSE, and UNEMP have unit root or not in the face of CSD’s presence, and its outcomes are reported in Table 4. The outcomes of the CIPS test disclose that BMI, INTUSE, MOBUSE, and UNEMP are non-stationary at their level values, but these series have become stationary following a first-differencing process.

The long-run interplay among BMI, INTUSE, MOBUSE, and UNEMP is analyzed by means of the LM bootstrap cointegration test, and the test statistics, together with the bootstrap and asymptotic *p*-values, are reported in Table 5. The asymptotic *p* values indicate the rejection of the H0 hypothesis of significant cointegration, while bootstrap *p* values suggest the acceptance of the H0 hypothesis. However, bootstrap *p* values are taken into account, resulting from the presence of CSD. Therefore, utilization of the LM cointegration test that is sensitive to CSD increases the robustness of our outcomes.

The long-run cointegration coefficients of INTUSE, MOBUSE, and UNEMP on obesity are estimated using the AMG estimator, and they are reported in Table 6. The results reveal that only unemployment negatively affects obesity at panel level. However, the countries’ cointegration coefficients uncover that Internet usage has a positive impact on obesity in Brazil, China, India, and South Africa, and mobile phone usage also has a positive effect on obesity in Brazil, China, India, and the Russian Federation. Furthermore, unemployment has a negative impact on obesity in China, the Russian Federation, and South Africa.

The AMG estimator results in Table 6 demonstrate that, at the panel level, unemployment exerts a negative and statistically significant effect on obesity. At the country level, Internet and mobile phone usage show positive and significant effects on obesity in most BRICS economies, except for the Russian Federation and South Africa. Although these coefficients are statistically significant, their magnitude suggests a limited practical effect.

Although the estimated coefficients for Internet and mobile phone usage are statistically significant, their magnitudes indicate a limited practical impact on obesity outcomes. These small effect sizes imply that while digitalization may correlate with changes in obesity prevalence, it is not a dominant explanatory factor. Instead, ICT use likely interacts with broader lifestyle and socio-economic variables, such as dietary patterns, urbanization, and income inequality, which jointly influence body-weight dynamics.

The heterogeneity observed across the BRICS countries suggests that the obesity–unemployment relationship is highly context-dependent. In China and the Russian Federation, the negative relationship may reflect a reduced caloric intake and limited access to processed foods among lower-income unemployed populations. In contrast, in Brazil and India, where informal employment is prevalent and food markets are more diverse, unemployment may have a weaker or even more positive effect on obesity. These variations indicate that the economic structure, social protection systems, and cultural dietary norms of each country mediate the unemployment–obesity nexus. Therefore, generalizing the results at the panel level should be approached with caution.

The causal association among body mass index (BMI), Internet usage (INTUSE), mobile phone use (MOBUSE), and unemployment (UNEMP) is investigated by the JKS non-causality test. The results, detailed in Table 7, provide robust evidence of bidirectional causality among BMI, INTUSE, and MOBUSE, indicating a reciprocal relationship where obesity and digital technology usage mutually influence each other over time.

## 5. Discussion

ICT indicators of Internet usage and mobile phone subscriptions can impact obesity through opposing channels based on the socio-economic development levels of individuals. Internet and mobile phone usage can help people to maintain a healthy life, because individuals can gain access to sources about healthy nutrition, diet, fitness, and physical activity at any time and anywhere [16,17,22]. Thus, Internet and mobile usage can cause people to avoid having obesity and obesity-related health problems. Nevertheless, improper usage of the Internet and mobile phones can contribute to obesity by means of unhealthy nutrition, physical inactivity, mental health problems, and low sleep quality [23,38,39]. Therefore, the net effect of Internet and mobile phone usage on obesity is theoretically ambiguous. Hence, the empirical literature on the association between Internet usage and obesity has remained inconclusive.

The variables examined—Internet usage, mobile phone subscriptions, and unemployment—affect obesity primarily through behavioral and socio-economic mechanisms. Increased Internet and mobile usage can reduce physical activity due to prolonged screen exposure, and may promote the online consumption of calorie-dense foods through targeted digital advertising. Conversely, these technologies can also enhance access to health-related information, nutrition tracking, and fitness applications, thereby fostering healthier lifestyles. The direction and intensity of their effects depend largely on users’ digital literacy, self-regulation, and socioeconomic context. Unemployment, in turn, influences obesity through both income and psychological channels: job loss reduces access to nutritious foods and healthcare, while psychological stress and lower self-esteem may increase physical inactivity, alcohol consumption, and irregular eating patterns.

On the one hand, Eliacik et al. [33], Ma and Sheng [35], Guo et al. [21], and Liu et al. [37] identified a positive impact of Internet usage on obesity, while Belojevic et al. [40], Souza et al. [24], Shi et al. [25], and Liu et al. [18] found a negative interplay between Internet usage and obesity. Our analysis indicates a positive association between Internet usage and obesity in all BRICS states, except the Russian Federation. In the associated empirical literature, Ma and Sheng [35], Deng et al. [36], and Guo et al. [21] analyzed the impact of Internet usage on obesity in China, and their results also indicate that Internet usage has a positive effect on obesity.

However, these results should be interpreted with caution. The analysis is based on macro-level panel data that capture statistical associations rather than individual-level causal relationships. Measurement limitations—including differences in data collection across countries and the use of aggregate ICT indicators—may influence the observed outcomes. Moreover, although the estimated coefficients for Internet and mobile phone usage are statistically significant, their magnitudes are relatively small, suggesting limited practical importance. Many unobserved factors, such as genetic predisposition, environmental context, and cultural habits also contribute to obesity, implying that ICT use is only one of several interacting determinants.

Our outcomes also indicate that mobile phones have a positive effect on obesity in BRICS states, except South Africa. On the other hand, Ma et al. [41], Akıncı et al. [20], Brodersen et al. [42], Andersen et al. [43], and Bagheri et al. [44] investigated the relationship between mobile phone usage and obesity in countries other than the BRICS states and uncovered a positive relationship between mobile phone use and obesity. However, Khokhar et al. [45], Liu et al. [46], Kim and Seo [47], and Pujia et al. [48] suggested that mobile phones were useful in weight loss in their research.

Last, unemployment can contribute to obesity through unhealthy nutrition, unhealthy lifestyle, increases in alcohol usage, smoking, and low sleeping quality in theoretical terms. In a similar vein, Latif et al. [49], Dietrich et al. [29], and Tobing [51] found a positive effect of unemployment on obesity. However, Hughes and Kumari [50] found a significant relationship between unemployment and being underweight, but a significant relationship between unemployment and obesity in nonsmokers in a UK sample. Contrary to the results of the limited empirical literature, our outcomes demonstrate that unemployment has a negative effect on obesity in China, the Russian Federation, and South Africa, in a similar vein to Hughes and Kumari [50]. Furthermore, Islam et al.’s [9] findings demonstrate that the prevalence of obesity was relatively higher among the people with higher income levels, and Ameye and Swinnen [10] also found that obesity was usually prevalent among rich people in low-income states and among the poor individuals in high-income states. Therefore, we evaluate that the negative effect of unemployment on obesity in China, the Russian Federation, and South Africa results from the inequalities in income distribution and current income level in these countries.

The causality analysis, based on the JKS test (Table 7), provides evidence of a bidirectional association between obesity and unemployment, suggesting that these two phenomena may reinforce each other over time. Specifically, higher unemployment may contribute to obesity through behavioral channels such as poor diet quality, psychological stress, and reduced access to healthcare, whereas obesity itself may also limit employment prospects and labor market participation.

This nuanced finding contrasts with much of the existing literature, which predominantly focuses on unidirectional effects. For example, Aydin and Aydin [27] explored the bilateral nexus between unemployment and obesity in the BRICS states and disclosed a significant causality between obesity and unemployment in China, highlighting obesity as a potential driver of a labor market disadvantage. On the other hand, Dietrich et al. [29] revealed that obesity was not a significant determinant of unemployment in Germany. Other studies such as Latif [49] and Tobing [51] reported a positive impact of unemployment on obesity, emphasizing the adverse consequences of job loss on health through mechanisms such as stress, reduced physical activity, and poorer dietary habits. Contrarily, Some et al. [53] and Groves and Wilcox [54] observed the negative effect of obesity on unemployment. More empirical studies are required to uncover the bidirectional relationship between unemployment and obesity.

## 6. Conclusions

Obesity continues to globally increase, and nearly 1.6 million people lose their life every year due to the noncommunicable diseases resulting from obesity. Furthermore, obesity has negative economic implications, including decreases in productivity and human capital and significant increases in health expenditures to cure obesity-related illnesses. Therefore, identifying the factors leading to obesity and the preventive and remedial measures to decrease obesity should be arranged based on these factors.

The limitations of the study are as follows.

This study focuses exclusively on the BRICS economies for the 1995–2022 period, and in turn, the generalizability of the findings to other developing or developed nations may be limited.

The main focus of the study is to investigate the short and long-term impact of Internet and mobile phone usage, along with unemployment, on adult obesity at country level, considering the remarkable increases in ICT penetration. Use of adult people with BMI ≥ 30 as a dependent variable, owing to the country-level analysis, leads us to disregard some factors, such as gender differences and childhood obesity, for the analysis. Additionally, other independent variables in the etiology of obesity are also disregarded.

This study investigates the impact of Internet and mobile phone usage and unemployment on adult obesity in the BRICS countries through country level analysis. Therefore, Internet usage and mobile cellular subscriptions, rather than prevalence of addiction, the utilization of health applications, and the extent of sedentary screen time, are obliged to be used, because these variables are not available on a country level. Furthermore, it is not possible to make an inference for an individual level, but the use of an AMG estimator enables us to make inferences about the effect of ICT indicators and unemployment on adult obesity for both the panel level and each country.

The consequences of the JKS causality test uncover a bidirectional interplay amongst adult obesity, Internet usage, mobile subscriptions, and unemployment. In other words, these variables affect each other in the short run. In addition, the panel cointegration coefficients forecast by the AMG estimator indicated that only unemployment has a negative effect on adult obesity in the BRICS states. Nevertheless, the cointegration coefficients of each BRICS state uncover that Internet usage negatively impacts adult obesity in Brazil, China, India, and South Africa, and mobile phone subscriptions positively affect obesity in Brazil, China, India, and the Russian Federation. Furthermore, unemployment has a negative impact on adult obesity in China, the Russian Federation, and South Africa. Our conclusions put forward that improper utilization of both the Internet and mobile phones can contribute to adult obesity. In addition, the negative impact of unemployment on adult obesity in China, the Russian Federation, and South Africa, differently from the limited empirical literature, is evaluated to have resulted from the current income level and inequalities in income distribution of these countries.

Given the complex interplay between digital technology, labor market dynamics, and public health outcomes, particularly obesity, a set of targeted policy strategies is necessary. One key area of focus is the promotion of digital health literacy. Public authorities should design and implement educational campaigns aimed at enhancing individuals’ understanding of how to use digital technologies responsibly, to support healthy lifestyles. Such campaigns should highlight how the Internet and mobile platforms can be leveraged to access information on nutrition, fitness, and mental well-being, while also cautioning against the risks of excessive screen time and physical inactivity.

Another important strategy involves leveraging digital platforms for health promotion. Governments and health institutions are encouraged to invest in the development and widespread dissemination of mobile and web-based applications that promote obesity prevention and weight management. These tools should be tailored to reach vulnerable and high-risk groups, including youth, and may be more impactful through strategic partnerships with private sector actors in the technology industry.

In addition, the study’s findings point to the need to address deeper structural issues in the labor market. The observed negative relationship between unemployment and obesity in certain BRICS countries may reflect broader socio-economic challenges, such as food insecurity and income inequality. In this context, labor market interventions should be complemented by robust social protection schemes and nutritional support programs to prevent undernutrition and ensure the health and well-being of unemployed individuals.

Policymakers should also consider integrating health perspectives into information and communication technology (ICT) development plans. As ICT infrastructure expands across BRICS nations, incorporating health impact assessments into digital policy frameworks will be crucial. This calls for coordinated efforts among various governmental departments—particularly those responsible for health, labor, education, and communication—to design cohesive strategies that mitigate potential health risks while maximizing the societal benefits of technological advancement.

Lastly, the variation in results across the BRICS countries reinforces the importance of context-sensitive policymaking. The effects of Internet and mobile phone usage and unemployment on obesity are not uniform across nations. Therefore, policy responses must be tailored to local conditions, accounting for socio-economic status, cultural habits, technological accessibility, and institutional capacities to effectively address obesity within specific national contexts.

Future research could explore the relationship between unemployment and obesity across countries with varying socio-economic development levels by incorporating micro-level data, examining gender- and age-specific effects, and analyzing moderating factors such as education, income inequality, and access to health services.

## Figures and Tables

**Table 1:** Definition of the variables.

Variables	Explanation	Source
BMI	Percentage of adults aged 18 and older with a BMI of 30 kg per square meter or higher	WHO [30]
INTUSE	Individuals using the Internet (% of population)	World Bank [31]
MOBUSE	Mobile cellular telephone subscriptions per 100 people	World Bank [32]
UNEMP	Unemployed people (% of total labor force)	World Bank [56]

**Table 2:** Descriptive statistics of BMI, INTUSE, MOBUSE, and UNEMP.

Variables	Mean Value	Std. Dev.	Min	Max
BMI	14.592	9.782	1.197	30.820
INTUSE	29.199	28.340	0.005	90.4
MOBUSE	70.741	57.082	0.008	170.14
UNEMP	10.734	7.540	3	34.007

**Table 3:** Outcomes of CSD and slope homogeneity tests.

CD Tests		Slope Homogeneity Tests	
Test	Test Statistic	Test	Test Statistic
LM	56.33 ***	Delta	13.413 ***
LM_adj._	27.14 ***	Bias-Adj. Delta	14.799 ***
LM CD	6.254 ***		

Note: *** significant at the 1% level.

**Table 4:** CIPS test outcomes.

Variables	Level		First Differenced Values	
	Constant	Constant + Trend	Constant	Constant + Trend
BMI	0.718	−0.452	−3.569 ***	−4.140
INTUSE	−0.033	−0.662	−3.297 ***	−3.152 ***
MOBUSE	−0.301	−0902	−3.485 ***	−3.532 ***
UNEMP	2.946	2.013	−5.964 ***	−5.285 ***

Note: *** significant at the 1% level.

**Table 5:** Outcomes of LM bootstrap cointegration test.

Constant			Constant + Trend		
LM Statistic	Bootstrap *p*-Value	Asymptotic *p*-Value	Test Statistic	Bootstrap *p*-Value	Asymptotic *p*-Value
17.545	0.440	0.000	10.021	0.214	0.000

Note: Bootstrap *p*-values are determined by means of 10,000 simulations, while asymptotic *p*-values are obtained from normal distribution.

**Table 6:** Cointegration coefficients by AMG estimator.

Countries	INTUSE	MOBUSE	UNEMP
Brazil	0.071 **	0.026 ***	−0.093
China	0.048 ***	0.034 ***	−0.597 ***
India	0.028 ***	0.005 ***	−0.003
Russian Federation	−0.007	0.009 ***	−0.060 ***
South Africa	0.059 ***	0.007	−0.256 ***
Panel	0.016	0.008	−0.202 *

Note: ***, **, * show the levels of significance at 1%, 5%, and 10%, respectively.

**Table 7:** JKS non-causality test’s outcomes.

H_0_ Hypothesis	HPJ Statistic
INTUSE ⇏ BMI	31.5266 ***
BMI ⇏ INTUSE	524.6703 ***
MOBUSE ⇏ BMI	9.3317 ***
BMI ⇏ MOBUSE	30.2740 ***
UNEMP ⇏ BMI	74.4726 ***
BMI ⇏ UNEMP	150.1843 ***

Note: *** significant at the 1%.

## Data Availability

The data presented in this study were derived from the following resources, available in the public domain: WHO at https://www.who.int/data/gho/data/themes/theme-details/GHO/gho-nutrition (accessed on 13 January 2025) and World Bank at https://data.worldbank.org/indicator/IT.NET.USER.ZS, https://data.worldbank.org/indicator/IT.CEL.SETS.P2, and https://data.worldbank.org/indicator/SL.UEM.TOTL.ZS (accessed on 13 January 2025).

## Abbreviations

The following abbreviations are used in this manuscript:

AMG	Augmented mean group
BRICS	Brazil, Russian Federation, India, China, and South Africa
CIPS	Cross-sectionally augmented Im–Pesaran–Shin
CSD	Cross-sectional dependence
ICT	Information and communication technologies
JKS	Juodis–Karavias–Sarafidis
LM	Lagrange multiplier
WHO	World Health Organization
